# Effects of Methanolic Extract Based-Gel From Saudi Pomegranate Peels With Enhanced Healing Potential on Excision Wounds in Diabetic Rats

**DOI:** 10.3389/fphar.2021.704503

**Published:** 2021-05-28

**Authors:** Shahid Karim, Huda M. Alkreathy, Aftab Ahmad, Mohammad Imran Khan

**Affiliations:** ^1^ Department of Pharmacology, Faculty of Medicine, King Abdulaziz University, Jeddah, Saudi Arabia; ^2^ Health Information Technology Department, Faculty of Applied Studies, King Abdulaziz University, Jeddah, Saudi Arabia; ^3^ Department of Biochemistry, Faculty of Science, King Abdulaziz University, Jeddah, Kingdom of Saudi Arabia; ^4^ Centre for Artificial Intelligence in Precision Medicine, King Abdulaziz University, Jeddah, Kingdom of Saudi Arabia

**Keywords:** pomegranate peel extract, diabetes, wound healing, topical gel, nitric oxide, VEGF, EGF, TGF beta 1

## Abstract

**Introduction:** Current study was designed to evaluate the wound healing activity of a Saudi pomegranate peel extract on excision wound healing in experimentally induced diabetes in rats.

**Methodology:** Animals were divided into three groups: diabetic excision wound with no treatment, diabetic excision wound with gel alone and diabetic excision wound with Saudi pomegranate peel extract in gel. Animals were monitored for clinical signs, weekly body weight, morbidity and mortality during entire study period. The efficacy parameters evaluated were percent wound contraction, Hydroxyproline content, estimation of Transforming Growth Factor ß1 (TGF-ß1), Vascular Endothelial Growth Factor (VEGF), and Epidermal Growth Factor (EGF) in wound lysates by ELISA, mRNA expression of TGF-ß1, VEGF, and EGF in wound lysates by qPCR, Estimation of nitric oxide (NO) and NO synthase (NOS) in Wound Lysates and histopathology of skin for reepithelization, neovascularization, and inflammation.

**Results:** The Saudi pomegranate peel extract in gel (5.0 g extract per 100 g gel) showed significant wound healing activity when compared to the vehicle control [*p* < 0.05] following 21 days of treatment. Animals in the control and treatment groups were apparently normal through the study with no significant differences in body weights between groups. Expression of mRNA of TGFβ1, EGF and VEGF in wounds was the highest on day 14 post treatment 4.3, 3.5 and 0.9 fold higher respectively in the treatment group when compared to vehicle control, and on day 21, the values were 0.12, 0.3 and 0.83, respectively. No statistically significant differences were observed in TGF-ß1 levels in wounds on days 4, 7, 14 and 21 post treatment when compared to the vehicle control (*p* > 0.05). Significantly higher levels of VEGF were observed in treatment group on day 7 and 21 when compared to vehicle control (*p* < 0.05). Significantly higher levels of EGF were observed in treatment group on day 7 and 21 when compared to vehicle control (*p* < 0.05). Mean hydroxyproline levels were higher in treatment group on days 4 and 7 when compared to vehicle control. NO levels in treatment group were significantly lower on days 7, 14 and 21 when compared to vehicle control (*p* < 0.05). NOS activity in treatment group were significantly lower on days 4 and 7 when compared to vehicle control (*p* < 0.05). Histopathological changes in skin wound in the treatment group were consistent with wound healing when compared to the vehicle group.

**Conclusion:** This study’s findings suggest that topical application of SPPE gel effectively enhanced wound healing in experimentally induced diabetic conditions.

## Introduction

Wound healing is a physiological response to repair injuries. Healing of wounds is a well-organized healing process characterized by haemostasis, cell migration, proliferation, angiogenesis, extracellular matrix deposition, wound contraction, and re-epithelialisation (Thu et al., 2012).

Diabetes mellitus is increasing at an unprecedented pace in Saudi Arabia, patients with type-2 diabetes (T2D) are often affected by delayed healing that develops into chronic wounds, diabetic foot ulcers, and may result in complications, including gangrene and limb amputation. These wound healing complications have an effect on both the quality of treatment and the cost of healthcare (Alshammary et al., 2020).

Evidence is available from many clinical and experimental studies of diabetic wounds that reveals that different phases of wound healing are impaired, such as delayed cell infiltration and granulation tissue formation, diminished angiogenesis, and impaired collagen formation (Coulombe, 2003; Brem and Tomic-Canic, 2007).

The process of delayed wound healing is multifactorial, with a protracted inflammatory period as well as deferred proliferation and remodeling stages (Qiu et al., 2007; Landén et al., 2016) discovered that diabetic patients with hyperglycemia had impaired cell proliferation and decreased collagen and growth factor production during wound healing. Reduced angiogenesis, re-epithelisation are also a major cause of impaired wound healing in diabetics with low levels of the growth factors like EGF, VEGF and TGF- ß1 (Costa and Soares, 2013). These biomarkers were chosen for this experimental study to assess drug mechanism during wound healing.

Various attempts have been made to improve wound healing in diabetics, but only a few successful clinical remedies are currently available. Alternative medicinal therapies based on natural ingredients are in high demand. The Holy Qur’an mentions that Pomegranate is a precious and prized fruit, and has been used for hundreds of years to prevent many illnesses and injuries. Pomegranate is a member of the Punicaceae family, Taif’s pomegranate is widely available across the Kingdom of Saudi Arabia. Pomegranate consists of various phytoconstituents having wide therapeutic potential (Reddy et al., 2007; Yuan et al., 2015). Most importantly its ability to treat inflammatory conditions, cancers, cardiovascular diseases, diabetes and dental problems (Lansky and Newman, 2007; Ismail et al., 2012; Darakhshan et al., 2019; Magangana et al., 2020). Almost all sections of a pomegranate have biological activities and are used in therapy. Pomegranate peel (skin, rind or husk), is distinguished by an internal arrangement of membranes, and it contains significant quantities of beneficial polyphenols, for example hydrolyzable tannins (ellagic tannins, punicalagins) (Murthy et al., 2004; Akhtar et al., 2015; Janarthanam and Sumathi, 2015). The medicinal benefits of pomegranate peel have been widely known, such as the use of aqueous pomegranate peel extract to treat dental plaque and aphthae. Indeed, over the past few decades, scientific experiments have laid a credible foundation for some of the common uses of the pomegranate peel. An initial study by Hayouni et al. (2011) reported the *in vivo* wound healing potential of a 5% (w/w) methanolic pomegranate peel extract-based ointment in guinea pigs. The formulation significantly increased wound contraction and epithelization, as determined by the mechanical, biochemical, and histopathological characteristics.

This study aims to investigate the effect of Saudi pomegranate peel extract (SPPE) on healing when used in experimentally induced, full thickness skin wounds in diabetic rats in terms of clinical (following wound formation, percent wound contraction until the 21st postoperative day; granulation tissue development; progress of epithelization; weekly body weight, morbidity and mortality), biochemical (Hydroxyproline, TGF-ß1, VEGF, EGF, NO and NOS) and histopathological (reepithelization, neovascularization, and inflammation) aspects.

## Experimental Procedures

### Materials

Pomegranate fruits of the *Taif region* were purchased from Hyperpanda Supermarkets, Jeddah, Saudi Arabia and authenticated at the Pharmacognosy Department, College of Pharmacy, King Saud University, Riyadh, Saudi Arabia. Manually the peel was stripped and cut into small bits. All of the chemicals and solvents used in the extraction and formulation processes were of analytical grade.

### Preparation of Extract

Saudi Pomegranate peel extract (SPPE) was prepared according to the method prescribed by (Shiban et al., 2012) with minor modifications. Prior to extraction with methanol (90%), mature pomegranate fruits (17.5 Brix) were manually peeled, washed, and sunshade dried. Briefly, finely powdered peels (50 g) were blended separately for 2 min (Waring blender) in 3,000 ml of 90% methanol. The mixture was then left at room temperature for 24 h in the dark before filtration (Whatman No. 1). To assess yields (%) per original materials, the extract was concentrated to dryness under reduced pressure at 40°C.

Thereafter, the semisolid methanolic extract was frozen at −70°C ± 10°C for 24 h inside of an ultra freezer (ColdLab, CL 600-80) for lyophilization (Terroni, model LS3000) for 26 h, and the resulting product was the lyophilized pomegranate peel extract, which was stored at −20°C in a hermetically sealed, light protected container (Zago et al., 2020).

### Estimation of Total Phenolics Content

It was estimated according of (Taga et al., 1984) method. 50 µL of aliquot sample (1 mg/ml) was mixed with 2.0 ml of 2% Na_2_CO_3_ and allowed to stand at room temperature for 2 min. Following incubation, 100 µL of 50% Folin Ciocalteau’s phenol reagent was added and the contents were thoroughly mixed and allowed to stand at room temperature for 30 min in the dark. The absorbance of all sample solutions was measured at 720 nm with a UV-visible spectrophotometer (Phenolic content is represented as mg gallic acid equivalents (GAE)/g of the extract).

### Estimation of Total Flavonoids Content

Total flavonoids content was determined based on the (Djeridane et al., 2006). One ml sample (1 mg/ml) was mixed with 1 ml of 2% AlCl_3_ solution. After 15 min of incubation at room temperature, the absorbance of the reaction mixture was measured at 430 nm due to the formation of a flavonoid–aluminium complex. Quercetin (0−100 μg/ml) was used as a standard to make the calibration curve. The amount of flavonoids was represented as quercetin equivalents (mg QE/g dry weight of the extract). All tests were carried out in triplicates.

### Preparation of SPPE Gel

In our lab, we produced SPPE and Vehicle gels. Briefly, the polyphenol gel was prepared with a base containing 5.0 g of carbopol 934 (LOBA chemicals, batch-L261782004), 51.5 ml of propylene glycol (SD fine chemicals batch-C18A/2018/2802/32), 0.5 ml of propylparaben (Central Drug House, batch-06117), 1.6 ml of triethanolamine, and distilled water was added in a quantity sufficient to prepare 100 g of Vehicle gel; while in the case of SPPE gel, 5.0 g of extract was added for every 100 g of total gel (Murthy et al., 2004). During the tests, this gel was put in collapsible tubes and stored in a cold, dry place.

### Experimental Animals

Seventy-eight female rats weighing between 180–200 g of Sprague Dawley strain were selected from Animal House at Theraindex Lifesciences, Bangalore, India, under pathogen-free conditions, they were kept in stainless steel wire cages. The rats were kept at a temperature of 18–22°C with a 12 h:12 h light/dark cycle and were given food and water as required during the experiment.

### Induction of Diabetes

Rats were fasted overnight. Following overnight fasting, diabetes was induced in animals by intraperitoneally injecting Alloxan monohydrate dissolved in physiological saline at a dose of 150 mg/kg body weight. Animals were allowed to drink 5% glucose solution overnight to overcome the drug induced hypoglycemia. Every animal’s fasting blood glucose levels were tested 72 h later using a commercially available glucometer (One Touch Ultra glucometer, Johnson & Johnson Co., United States) to confirm diabetes induction (Blood glucose greater than 250 mg/dl). Animals who had successfully been induced with diabetes were chosen and grouped for further research.

### Excision Wound Creation

The dorsal skin of animals was shaved with a pet trimmer 5 days after diabetes induction. An intraperitoneal injection of ketamine (70 mg/kg) + xylazine (10 mg/kg) was used to anesthetize the animals. A 2 cm × 2 cm wound was made on the animal’s depilated dorsal thoracic region. Under anaesthesia, a predetermined region of 2 cm × 2cm skin was excised under aseptic conditions using autoclaved surgical instruments. To alleviate pain and tension following excision, 5 ml/kg ketoprofen was administered subcutaneously. Following the development of the wounds, the animals were housed individually with species-specific enrichment. The wound area was determined on the day of creation by tracing the wound boundaries on a transparent sheet of paper. Animals were treated with vehicle and test formulations for 21 days after the wound was developed, as stated in the experimental design (Table 1).

**TABLE 1:** Experimental study design.

Group	Group ID	Treatment, (number of days)	No. of animals to be sacrificed post wound creation for collection of skin samples					Total no. of animals/group
			Day 0^a^	Day 4^a^	Day 7^a^	Day14^a^	Day 21^a^	
1	Diabetic control (no treatment)	No treatment (21 days)	6	6	6	6	6	30
2	Vehicle (diabetic excision wound with gel alone)	Topical, twice daily (21 days)	-	6	6	6	6	24
3	SPPE gel [diabetic excision wound with SPPE in gel (5.0 g extract per 100 g gel)]	Topical, twice daily, (21 days)	-	6	6	6	6	24

a All samples for HP analysis, hydroxyproline content, TGF-ß1, VEGF, EGF (ELISA and qPCR (pooled analysis)); NO, NO synthase.

#### Treatment

Animals were treated as shown in the experimental design, by topical application, twice daily for a period of 21 days.

### Clinical Observations

#### Body Weights

Body weights were recorded on the day of diabetes induction, before and after randomization and weekly during treatment period.

#### Clinical Signs

Animals were observed for clinical signs daily during the entire experimental period.

### Measurement of Wound Contraction

Wounds were assessed planimetrically on days 0, 4, 7, 14 and 21 by following the progressive changes in wound area. The size of wound was traced on transparent paper and placed on graph paper to estimate the areas. The estimated surface area was used to calculate the percentage of wound contraction, taking initial size of wound, 200 mm^2^ as 100%, using the following formula:

$$\%\;\text{wound}\;\text{contraction}=\;\frac{\text{Initial}\;\text{wound}\;\text{size}-\text{Specific}\;\text{day}\;\text{wound}\;\text{size}}{\text{Initial}\;\text{wound}\;\text{size}}\times 100$$

### Collection of Skin Samples

On days 4, 7, 14, and 21 post-wound creation and during treatment, six rats per group were sacrificed with overdose of CO_2_. The tissue adjacent to the wound was excised down to the fascia and divided into three pieces through the least healed portion. One piece of the wound was weighed, homogenized in saline, and stored in liquid nitrogen for biomarker studies; the second piece of wound was weighed and kept in oven for drying and hydroxyproline assay; and third piece was fixed in 4% paraformaldehyde for histological examination.

### Histological Analysis

Skin samples obtained on days 4, 7, 14, and 21 were processed and paraffin blocks were prepared. The blocks were sectioned to 3 to 5 microns and placed on clean glass slides using a rotatory microtome. Hematoxylin and eosin (H&E) dye was used to stain the slides, which were then examined under a light microscope. Each slide was graded on a 4-point scale for fibroblast proliferation, collagen formation, neovascularization, granulation tissue, and epithelialization: 0 = none, 1 = rare or minimal, 2 = moderate, 3 = abundant, and 4 = severe or marked. Three separate sections were chosen at random from each specimen for histological evaluation.

### Determination of Hydroxyproline Content in Wound Lysates

Wound tissues were dried at 60°C for 24 h and weighed, homogenized in PBS and used for determination of hydroxyproline content. Hydroxyproline was measured in wound skin lysates using a rat Hydroxyproline enzyme-linked immunosorbent assay kit (Rat Hydroxyproline (Hyp) ELISA Cat# K11-0512). The concentrations of Hydroxyproline were normalized to total protein content using BCA protein assay kit.

### Estimation of TGF-ß1, VEGF, and EGF in Wound Lysates

Rat tissue homogenate samples were used to determine TGF-β1 (Rat Transforming Growth Factor Beta 1 (TGFβ1) GENLISA™ ELISA Ref# KLR1688), EGF (Rat Endocrine Gland Vascular Endothelial Growth Factor (EG-VEGF) GENLISA™ ELISA Ref# KLR0461), and VEGF (Rat Vascular Endothelial Cell Growth Factor (VEGF/VEGF-A) GENLISA™ ELISA Ref# KLR0659) levels by ELISA. Homogenate samples were diluted and assayed according to the manufacturer’s instructions and analyzed on a TECAN-Nano Quant Plate™ system.

### Quantitative Real-Time PCR for mRNA Expression of TGF-ß1, VEGF, and EGF in Wound Lysates

Rat tissue was added to 1 ml of PBS and homogenised. RNA was isolated from 500 µl of homogenate by using the Nucleo-pore® RNASure® Mini Kit (Genetix: NP-84105) and used in a quantitative real-time PCR assay with the RNA-direct™ SYBR® Green Realtime PCR Master Mix (Toyobo: QRT-201). The q-RT-PCR was done on a QuantStudio™ 3 Real-Time PCR System.

Expression levels of TGF-ß1, VEGF, and EGF in wound tissues of rats in each group was also estimated using pooled samples per group by one-step SYBR Green Quantitative Real-time PCR (qRT-PCR) (to see relative quantification by fold change) to confirm the ELISA assay results.

### Estimation of Nitric Oxide (NO) and NO Synthase (NOS) in Wound Lysates

Nitric Oxide and Nitric Oxide synthase levels in homogenates were measured by a colorimetric assay using Universal Biologicals kits (QuantiChrom™ Nitric Oxide Assay Kit D2NO-100 and EnzyChrom™ Nitric Oxide synthase Assay Kit (ENOS-100) ENOS-100) and analyzed on a TECAN-NanoQuant Plate™ system.

## Statistical Analysis

All values are presented as mean ± SEM. Differences were considered to be significant at *p* < 0.05. One-way analysis of variance (ANOVA) was used to determine differences between time points. The differences between control and treatment groups were compared by independent sample *t*-test. Kruskal-Wallis test was used for analysing semi-quantitative HP scores. The Graphpad Prism (v 5.0) software was used for statistical analysis.

## Ethical Aspects

This study was performed as per ethical practices laid down in the CPCSEA guidelines (2003) for animal care and use. The study was approved by the Institutional Animals Ethics Committee (IAEC) of the test facility. IAEC/15/2020/187.

## Results

### Yield, Total Phenolic and Flavonoid Content of Extract

**FIGURE 1:**
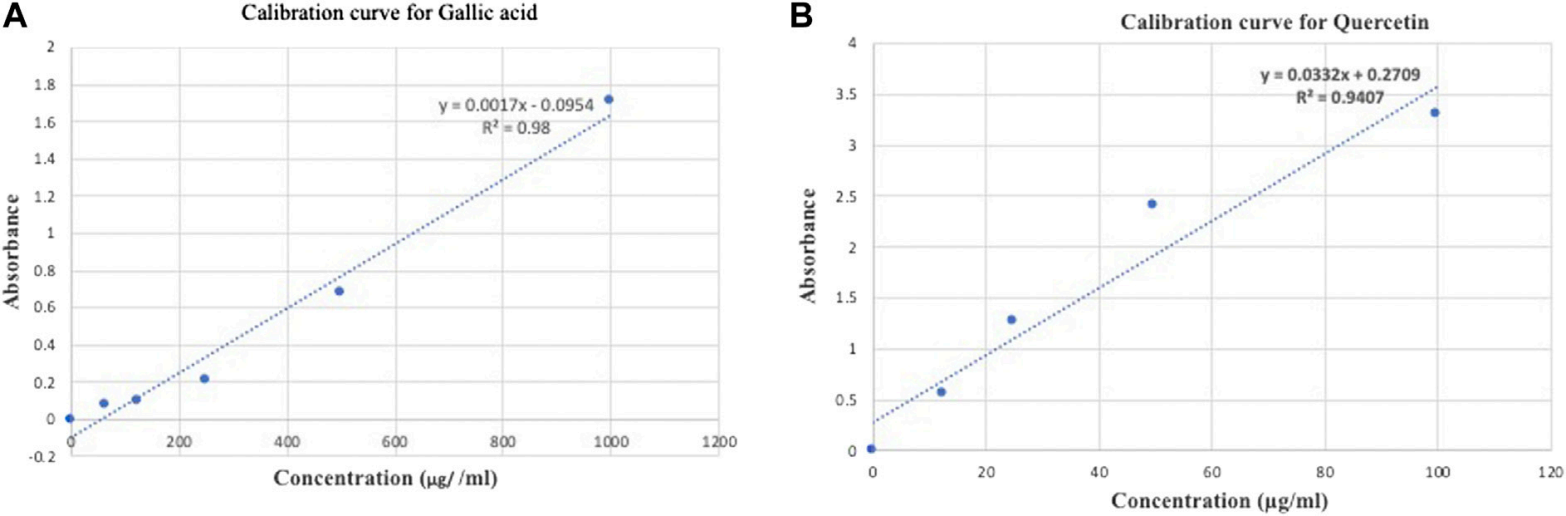
**(A)** Calibration curve of standard gallic acid for determination of total phenolic content in SPPE. **(B)** Calibration curve of standard quercetin for determination of total flavonoid content in SPPE.

The methanolic extract yield was 14.5% w/w of pomegranate peel. The result of total phenolic content was calculated from the regression equation of the standard plot (y = 0.0017x − 0.0954, r2 = 0.98) (Figure 1A), and is expressed as gallic acid equivalent. Flavonoid content was calculated from the regression equation of the standard plot (y = 0.00332x + 0.02709, r2 = 0.9407) and is expressed as quercetin equivalents (QE) (Figure 1B). The total phenolic contents were 282.15 ± 0.04 mg GAE/g of SPPE while total flavonoids contents mg QAE/gm of dry powder was 7.25 ± 0.01 mg/gm of SPPE.

### Wound Contraction

**FIGURE 2:**
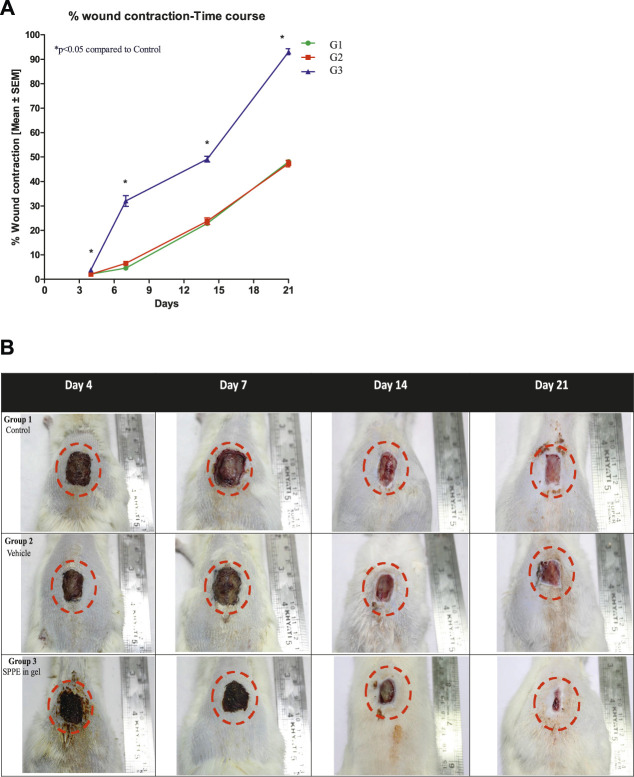
Effect of SPPE gel on wound contraction in diabetic rats. **(A)** The percentage wound contraction was measured at 4, 7, 14, and 21 days after wound creation. Data expressed as mean ± SEM (*n* = 6). **(B)** Representative pictures of wound contraction at 4, 7, 14, and 21 days after wound creation.

The SPPE gel treated group (Group 3) showed significantly higher wound contraction when compared to the diabetic control (Group 1) on days 4, 7, 14 and 21 (Figure 2A). As observed in Figure 2B, on day 21, the wound closure rates of diabetic rats in group 3 were over 90% while in group 1 and vehicle group (Group 2) it was 48 and 47%, respectively. On days 4, 7 and 14 the wound closure rate of diabetic rats in the SPPE gel group was significantly higher than that of rats in the group 1. Group 2 showed no significant change on the wound closure rate as compared to Group 1.

### Clinical Observations

No mortality occurred during the course of experiment. Animals in all the groups were apparently normal through the study, there was no significant loss in body weight through the study period (Table 2). No clinical signs were observed in any of the animals during the course of experiment except for minor discomfort during the first week of experiment, which could be ascribed to wound creation.

**TABLE 2:** Effect of SPPE gel on body weight in diabetic rats.

Group no.	Treatment	Body weight (g), mean ± SEM				
		Day 0	Day 4	Day 7	Day 14	Day 21
1	Diabetic control (no treatment)	182.9 ± 2.28	173.42 ± 2.37	171.06 ± 2.44	170.42 ± 3.21	164.5 ± 3.71
2	Vehicle (diabetic excision wound with gel alone)	182.5 ± 2.81	173.59 ± 1.91	165.89 ± 2.29	164.17 ± 2.86	167.5 ± 3.51
3	SPPE gel [diabetic excision wound with SPPE in gel (5.0 g extract per 100 g gel)]	182.59 ± 2.21	176.88 ± 1.88	166.78 ± 2.35	169 ± 1.9	172.17 ± 2.11

The body weight was measured at 4, 7, 14, and 21 days after wound creation. Values are represented as mean ± SEM (*n* = 6).

### Effect of SPPE on Hydroxyproline Content in Wound Lysates

**FIGURE 3:**
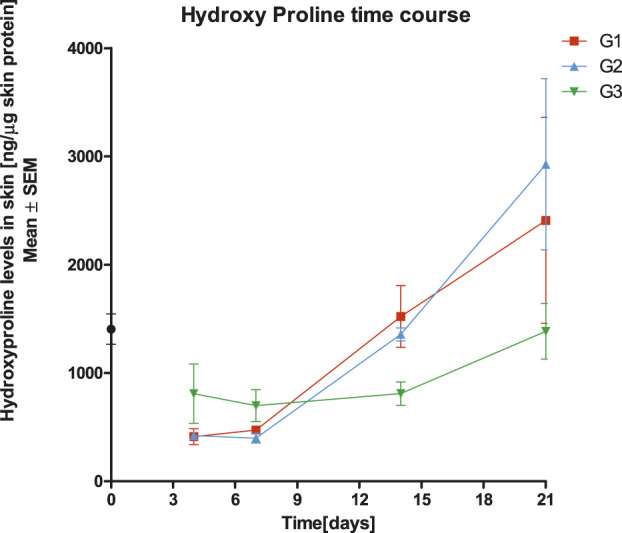
Effect of treatments with Diabetic control (G1), Vehicle (G2), and SPPE gel (G3) on hydroxyproline contents in wound tissues of rats at day 4, 7, 14 and 21 post wounding. Values are represented as mean ± SEM (*n* = 6).

The amount of hydroxyproline in wound tissue is widely used as a measure of collagen quality. As shown in Figure 3, the mean hydroxyproline levels were higher in G3 (Diabetic Excision Wound with SPPE gel) on days 4 and 7 when compared to G2 (Diabetic Excision Wound with gel alone). No statistically significant differences were observed in hydroxyproline levels on days 4, 7, 14 and 21 in G3 post treatment when compared to the vehicle control G2 (*p* > 0.05). So far, the results indicate that SPPE gel raises collagen content in diabetic wounds (Figure 3).

### Effect of SPPE Gel on Expression of TGF-β1, VEGF, and EGF

**FIGURE 4:**
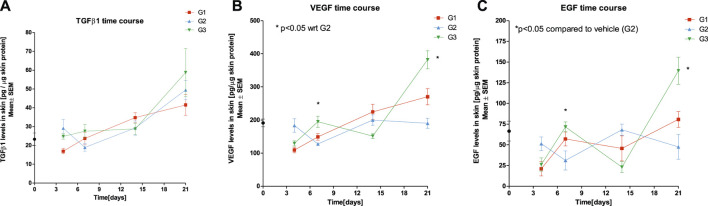
Effect of SPPE gel on expression of TGF-β1 **(A)**, VEGF **(B)**, and EGF **(C)** in wound tissues of rats at day 4, 7, 14, and 21 post treatment Diabetic control (G1), Vehicle (G2), SPPE gel (G3). Values are represented as mean ± SEM (*n* = 6).

There were higher levels of TGF-ß1 in G3 when compared to G1 and on days 7 and 21, post treatment. No statistically significant differences were observed in TGF-ß1 levels on days 4, 7, 14 and 21 post treatment when compared to the vehicle control G2 (*p* > 0.05). Significantly higher levels of VEGF and EGF were observed in G3 on day 7 and 21 when compared to vehicle control G2 (*p* < 0.05) (Figure 4).

The expression of TGFβ1, EGF and VEGF was the highest on day 14 post-treatment 4.3, 3.5 and 0.9 fold higher in G3 when compared to G2, and on Day 21, the values were 0.12, 0.3 and 0.83 respectively (Table 3).

**TABLE 3:** Fold change in gene expression in SPPE gel (G3) relative to vehicle group (G2) through treatment.

Gene	Fold change in G3 relative to G2			
	Time (day)			
	4	7	14	21
TGFβ1	45.98	0.00	4.26	0.12
EGF	0.00	0.28	3.52	0.29
VEGF	0.39	0.02	0.88	0.83

### Effect of SPPE Gel on NO Production and NOS Activity

**FIGURE 5:**
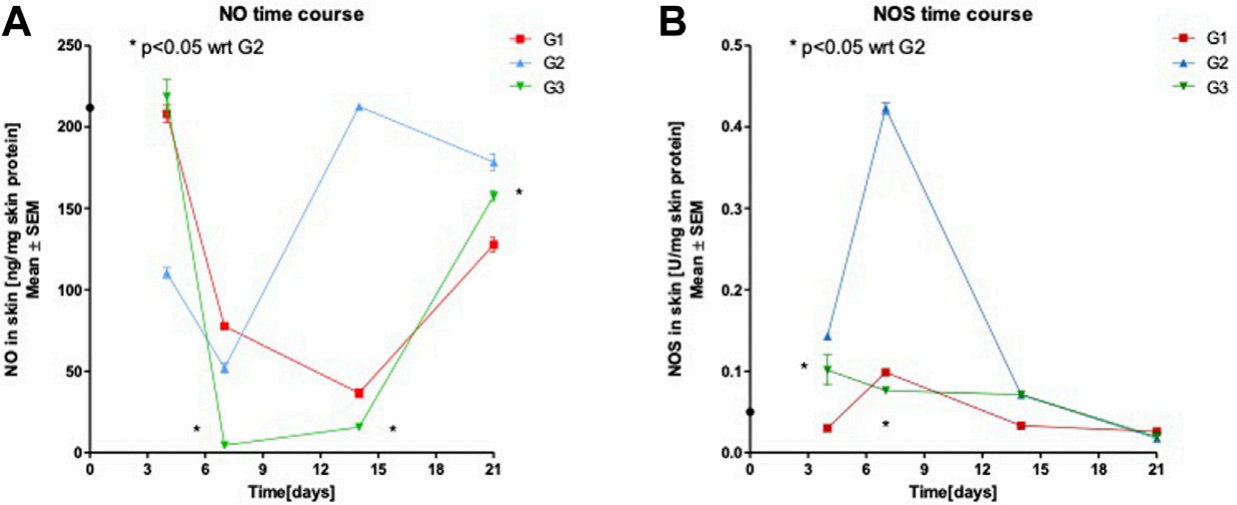
Nitric Oxide (NO) levels **(A)** and Nitric Oxide synthase (NOS) activity **(B)** in skin homogenate following treatments with diabetic control (G1), Vehicle (G2), SPPE gel (G3) Values are represented as mean ± SEM (*n* = 6).

As shown in Figure 5A, the NO levels in SPPE treated rats were significantly lower on days 7, 14 and 21 when compared to Vehicle group (*p* < 0.05). While in Figure 5B, the NOS activity in the wound tissues of the SPPE gel group were significantly lower on days 4 and 7 when compared to G2 (*p* < 0.05). The gel Vehicle showed no significant effect on NO production and NOS activity in the wound tissues of diabetic rats.

### Histological Evaluations

**FIGURE 6:**
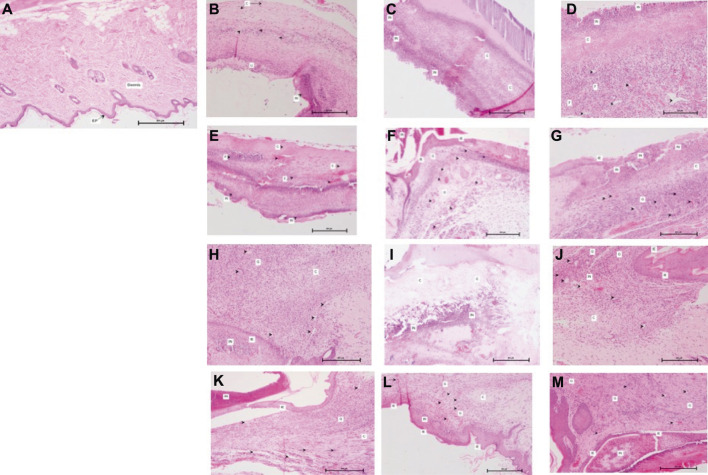
**(A)** G1, Day 0; Skin with normal epidermis (EP) and dermis (H&E X 100×). **(B)** G1, day 4: Skin with inflammatory cells (IN), collagen formation (C), neovascularization (arrowhead) and ulceration (U) (H&E X 100×). **(C)** G2, Day 4: Skin with inflammatory cells (IN) and collagen formation (C) (H&E X 100×) **(D)** G3, Day 4: Skin with inflammatory cells (IN), collagen formation (C) and fibroblast proliferation (F) and neovascularization (arrowhead) (H&E X 100×). **(E)** G1, Day 7: Skin with inflammatory cells (IN), collagen formation (C), fibroblast proliferation (F) and neovascularization (arrowhead) (H&E X 100×). **(F)** G2, day 7: Skin with inflammatory cells (IN), granulation tissue (G), fibroblast proliferation (arrow), neovascularization (arrowhead) and immature epidermis (IE) (H&E X 100×). **(G)** G3, Day 7: Skin with inflammatory cells (IN), collagen formation (C), granulation tissue (G), fibroblast proliferation (arrow), neovascularization (arrowhead) and immature epidermis (IE) (H&E X 100×). **(H)** G1, Day 14: Skin with inflammatory cells (IN), collagen formation (C), neovascularization (arrowhead) and immature epidermis (IE) (H&E X 100×). **(I)** G2, Day 14: Skin with inflammatory cells (IN) and collagen formation (C) (H&E X 100×). **(J)** G3, Day 14: Skin with inflammatory cells (IN), collagen formation (C), granulation tissue (G), fibroblast proliferation (arrow), neovascularization (arrowhead) and immature epidermis (IE) (H&E X 100×). **(K)** G1, Day 21: Skin with inflammatory cells (IN), collagen formation (C), granulation tissue (G), fibroblast proliferation (arrow), neovascularization (arrowhead) and immature epidermis (IE) (H&E X 100×). **(L)** G2*,* Day 21: Skin with inflammatory cells (IN), collagen formation (C), granulation tissue (G), fibroblast proliferation (arrow), neovascularization (arrowhead) and immature epidermis (IE) (H&E X 100×). **(M)** G3, Day 21: Skin with inflammatory cells (IN), granulation tissue (G), neovascularization (arrowhead) and immature epidermis (IE) (H&E X 100×).

**FIGURE 7:**
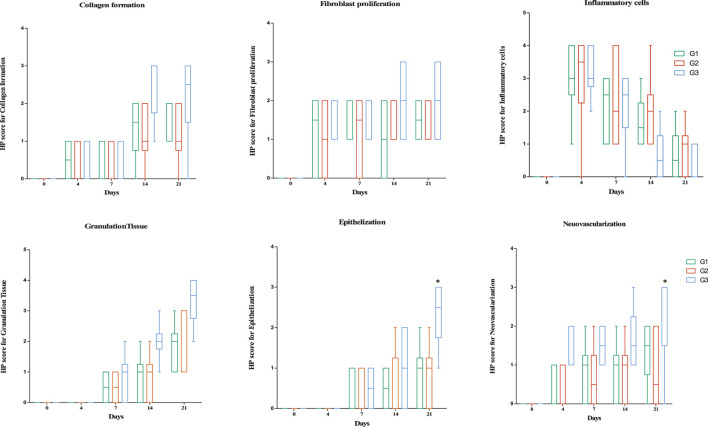
Box plots of histopathology scores for skin parameters through treatment. Horizontal lines in boxes represent median and error bars represent minimum and maximum. *Significantly different from G2 [*p* < 0.05, Kruskal-Wallis test]. 0 = None, 1 = Rare or Minimal, 2 = Moderate, 3 = Abundant, and 4 = Severe or Marked.

Histological examination determined the degree of fibroblast infiltration, collagen regeneration, vascularization, and epithelialization in the wound area. In the wounds of rats in the G1 (Day 0), the granulation tissue was thin and few vessels, fibroblasts, and collagen fibers were distributed in the incisional space. After the rats in the SPPE gel group were treated with SPPE gel, healing of diabetic wounds markedly improved, as indicated by the histological scores in Figures 6, 7. Compared with diabetic rats, a marked increase in fibroblast formation in the SPPE gel-treated diabetic rats was observed 4 days post-wounding. Collagen regeneration was more abundant in the Group 3 than that in the Group 1 and 2 at on day 14 post-wounding. Furthermore, higher vascularization was observed in the Group 3 on days 4,7 and 14 post-wounding. Epithelialization and granulation were also significantly greater in the Group 3 than that in the Group 1 and Group 2 on day 21 (Figures 6 and 7).

## Discussion

Pomegranate Peel is an excellent source of organic acids and a wide range of other nutrients that contribute to market popularity and demand (Singh et al., 2018; Andrade et al., 2019; Pirzadeh et al., 2021). Plant metabolites present in pomegranate peel like phenolic acids and flavonoids have an active role in wound healing (Fleck et al., 2016; Han and Ceilley, 2017; AlMatar et al., 2018; Wang et al., 2018). In the current study the total phenolic content of the extract was in the range of the average (210.36 mg gallic acid equivalent/g) experimental phenolic yield value under the optimum conditions as reported earlier (Zheng et al., 2011; Calín-Sánchez et al., 2013; John et al., 2017; Kaur et al., 2018).

Many studies have been performed with the objective of accelerating wound healing in diabetics (Murthy et al., 2004; Ma et al., 2015; Dughayshim Alshammari et al., 2017; Ragab et al., 2019). Nonetheless, there are limited work done on the effects of Pomegranate peel of Saudi origin on diabetic wound healing.

We found that SPPE gel could improve the healing of excision wounds in alloxan-induced diabetic rats. When diabetic rats' wounds were treated with SPPE gel, wound closure time was significantly reduced. Similar findings were reported by (Dughayshim Alshammari et al., 2017) where topical application of Saudi *pomegranate rind powder* enhanced the healing of excision wounds in rats through improvements in wound contraction and reductions in wound size.

Wound closure is a complex and well-coordinated association of cells, extracellular matrix, and cytokines. Fibroblasts are the key cells involved in wound healing. Fibroblast migration and proliferation are needed for extracellular matrix secretion. Collagen, a major extracellular matrix protein, is the component that ultimately contributes to wound strength (Gurtner et al., 2008; Demidova-Rice et al., 2012). In our research (Figure 6), histological analysis revealed increased fibroblast proliferation and collagen regeneration are one of the earliest events in diabetic rats' wound tissues treated with SPPE. Fibroblast are the key cells in granulated tissues and its function in rat skin excision wounds has been reported (Spyrou et al., 1998).

Granulation tissue consists of various cells, and vascular capillaries and loose connective tissues in its stroma are usually produced to fill up the injured space (Muniandy et al., 2018). Figure 5M also suggests accelerated wound healing through the formation of granulation tissues on diabetic wound tissue of rats treated with SPPE gel.

It has been documented that the amount of hydroxyproline in wound tissue is widely used as a measure of collagen quality (Boakye et al., 2018). To validate our histological findings, hydroxyproline contents in the lesions were assessed, and the results revealed that hydroxyproline contents in the wound tissues of SPPE-treated rats were higher. These findings were consistent with (Yan et al., 2013) who reported an increased hydroxyproline levels in wounds of diabetic rats treated with pomegranate peel polyphenol gel.

The expression of TGF- β 1 in wound tissues was assessed in order to better understand the mechanism of SPPE gel in increasing collagen content. TGF- β 1 is the most common isoform in most tissues, and it is particularly abundant in platelets. 18 TGF- β 1 is critical in mediating collagen synthesis and degradation. TGF-1 has been shown in studies to facilitate fibroblast migration and proliferation during wound healing (Bitar and Labbad, 1996). Exogenous TGF- β 1 administration could increase the amount of collagen in diabetic rats' wounds (Costa and Soares, 2013).

Thus, rising endogenous growth factor release is a possible mechanism of wound tissue repair. On day 14 post-wounding, ELISA assays revealed that TGF- β 1 expression in the wound tissue of rats in the SPPE gel group was higher than that in Vehicle treated group; these findings were verified by qRT-PCR results. Upregulation of TGF-B1 was also reported by Tan and co-workers in diabetic rats treated with VCN-2 film (Tan et al., 2019).

SPPE gel has been shown to accelerate neovascularization in the wound tissues of diabetic rats in histological studies. Neovascularization throughout wound healing provides an effective means by supplying vital nutrients and oxygen to the wound site while also promoting the formation of granulation tissue (Tonnesen et al., 2000). To fully understand how SPPE gel increases neovascularization, we determined the expression of VEGF in wound tissues. Pathological phenomena such as tissue repair, which includes neovascularization and altered vascular permeability, tend to be influenced by VEGF. VEGF promotes angiogenesis amid wound healing by causing endothelial cells to migrate into the extracellular matrix. On days 4, 7, 14 and 21 after wounding, ELISA assays revealed that VEGF expression in wound tissues of rats in the SPPE gel was significantly higher than that in diabetic rats; these findings are also supported by qRT-PCR results. These findings are in accordance with earlier studies (Kant et al., 2015; Zhou et al., 2017).

Histological evidence (Figure 6) also supports SPPE gel’s ability to accelerate epithelialization in diabetic rat wound tissues. Re-epithelialization, which includes the proliferation, migration, and differentiation of keratinocytes from the wound margins, is an essential process during the early stages of healing (Okonkwo et al., 2020).

EGF, which promotes epithelialization, is secreted by platelets, keratinocytes, and macrophages during the wound healing process. EGF activates epithelial cell mitosis and chemotaxis, which leads to epithelialization (Demidova-Rice et al., 2012). Furthermore, EGF has been shown to affect wound healing by increasing fibronectin synthesis (Barrientos et al., 2008; Bodnar et al., 2016).

We have studied expression of EGF in wound tissue using ELISA based method and found that on day 7 after the wounding, there was a considerably higher EGF content in the rats treated with SPPE gel than that of the diabetic group. Similarly, qRT-PCR findings showed that the mRNA expression of EGF in wound tissues from rats in the SPPE-treated diabetic group was significantly higher than in rats in the vehicle control group 7and 21 days after wounding. These findings are in confirmation with the earlier reports of Yan and co-workers (Yan et al., 2013).

Recent studies show that NO plays a significant role in normal wound healing. Nitric oxide deficiency has been identified as a key mechanism underlying delayed healing in diabetic ulcers (Hayashi et al., 2012). NO facilitates wound healing processes such as angiogenesis, migration, and proliferation of fibroblasts, epithelial cells, endothelial cells, and keratinocytes, but it also plays a crucial role in intercellular communication to regulate cell proliferation, collagen production, and wound healing (Witte and Barbul, 2002).

Endothelial NOS (eNOS) and neuronal NOS (nNOS) are constitutive NOS isoforms, and one inducible NOS isoform produces NO (iNOS). NO can be generated by a variety of cells in a wound. Platelets, macrophages, fibroblasts, endothelial cells, and keratinocytes are examples of these cells (Luo and Chen, 2005; Ni et al., 2010). The use of wound fluid nitrate quantification as a measure of wound NO bioactivity in preliminary clinical wound healing studies suggests that threshold values of wound fluid nitrate can be a useful diagnostic parameter of good wound healing in Diabetic Foot Ulcer patients (Boykin, 2010). An optimal release of NO would improve wound healing, however over-production of NO over time can result in the development of chronic wounds (Soneja et al., 2005).

NO levels in SPPE treated rats were significantly lower than the vehicle group throughout the treatment period, and NOS activity in the wound tissues of SPPE treated rats was significantly lower till Day 7 than in the wound tissues of vehicle treated rats. These current findings for NOS activity with SPPE gel can enhance the healing of excisional wounds in diabetic rats are consistent with the findings of Tan and co-workers (Tan et al., 2019) Vicenin-2 hydrocolloid film treatment in diabetic excisional wound in mice.

In conclusion, the current findings suggest that SPPE gel can increase fibroblast proliferation, neovascularization, granulation tissue, epithelialization, and collagen deposition, as well as enhance excisional wound healing in diabetic rats. SPPE’s mechanism on diabetic injuries can be due to its role in upregulating the content of hydroxyproline, TGF-β1, VEGF and EGP expressions in wounded tissues, an optimum NO production, and NOS activity.

Further studies would be required to see the influence of SPPE gel on inflammation and antibacterial effects in diabetic models.

## Data Availability

The original contributions presented in the study are included in the article/Supplementary Material, further inquiries can be directed to the corresponding author.
